# Study on Major Parasitic Diseases of Adult Honeybees in Three Districts of Kaffa Zone, Southern Ethiopia

**DOI:** 10.1155/2021/6346703

**Published:** 2021-08-11

**Authors:** Semere Solomon, Tadesse Degu, Haben Fesseha, Mesfin Mathewos

**Affiliations:** ^**1**^ Shishonde District Veterinary Clinic, Kaffa Zone, Bonga, Ethiopia; ^2^Raya Kobo District Veterinary Clinic, North Wollo Zone, Kobo, Ethiopia; ^3^School of Veterinary Medicine, Wolaita Sodo University, P.O. Box 138, Wolaita Sodo, Ethiopia

## Abstract

**Results:**

Out of 384 samples, the overall prevalence of small hive beetle, *Nosema apis*, and amoeba *(Malpighamoeba mellificae)* diseases was 39%, 45.3%, and 40.3%, respectively. The overall prevalence of these three diseases was ascertained, with a statistically significant (*p* < 0.05) variation in the overall prevalence of *N. apis* disease and amoeba (*M. mellificae*) disease between hive type and agroecologies.

**Conclusion:**

The research indicates that *N. apis*, *M. mellificae*, and small hive beetle are the major parasites that affect honeybees in the study area. In order to classify and describe honeybee diseases and pests associated with Ethiopia's local honeybees, several field diagnostic surveys and laboratory research works need to be performed.

## 1. Introduction

With wide climatic and unique flowering plants, Ethiopia is among the highly suitable countries for beekeeping. To this effect, the country sustains a large number of bee colonies with the long-established practice of beekeeping. The country sustains about 10 million bee colonies annually producing 53,000 tons of honey per annum, thus sharing 23.5% of Africa's and 2.35% of the world's honey production [1]. This makes the country rank 1^st^ in the continent and 10^th^ in the world. Besides, the beeswax production in Ethiopia is about 4,300 tons, which makes the country rank first in Africa and fourth in the world [2–4]. The same source also indicated that about 1.8 million farmers are engaged in beekeeping activities with annual productivities of modern or box hives up to 50 kg of honey [5].

The beekeeping industry has undergone a vital revolution in the past few years. The importance of honey as food, the need for honeybee products for industrial uses, and the increment of efficient agricultural crop production through honeybee pollination have evoked changes in location, sources, and movement of bees and bees' products. As a result, there are new threats for the spread and introduction of bee diseases, pests into areas where they were not known before. The world distribution of honeybee diseases, pests, and predators are of great importance to beekeepers. This is because if it once occurs in the colony, they cause partial or total loss of colonies and most of them spread very quickly and are difficult to treat [6].

The essential and valuable contributions of honeybees depend upon the healthy population of honeybees [7]. The health of honeybees has been one of the most important topics in apiculture research in recent years [8]. This is mainly associated with the recent emergence of high honeybee colony losses in many parts of the world [8, 9] and the vulnerability of honeybees to parasitic mites, fungi, *Protozoa*, viruses, and bacteria [10]. These pathogens and parasites can have harmful effects on honeybee health and the services they offer, which in turn can lead to severe economic losses [2, 10].

Like all other living organisms, honeybees can be infested with harmful diseases and pests. Hence, conducting regular surveys and recognizing prevalent disease conditions are the key steps to protect honeybee colonies and their products from harmful diseases and pests [11, 12]. Transmission of bee diseases from one colony to another can be by adult bees, reuse of contaminated comb, beekeepers, beekeeping equipment, feeding of infected honey, and pollen [13–15].

The agroecology of Ethiopia is not only favorable to honeybees but also to different kinds of honeybee pests and predators that are interacting with the life of honeybees [16]. The most commonly known honeybee diseases and pests reported to exist in Ethiopia are *Nosema apis* and *Malpighamoeba mellificae* [17, 18]; small hive beetles (*Aethina tumida* Murray; Coleoptera: Nitidulidae) have been commonly implicated; however, the evidence on the magnitude and distribution of pests and diseases is still not explored well [19, 20]. The seriousness of honeybee diseases and pests indeed differs within or among colonies, apiaries, areas, and weather conditions [21].

Nosema disease that is caused by a protozoan known as *N. apis* is known to weaken the bee colonies by infecting the intestinal tract of adult bees and causing the death of the bees several days earlier than the normal healthy one by 22–44%. *N. apis* causes detrimental effects on honeybees, colony development, queen performance, and honey production [22]. The existence of this disease in Ethiopia was reported in 1989 through preliminary laboratory diagnosis on 38 bee colonies at Holeta Bee Research Center (HBRC) [18].

Amoeba disease is a disease of honeybees caused by a single-celled parasite called *M. mellificae.* The parasite affects the Malpighian tubules of honeybees and shortens their life cycle [22]. Together with *N. apis*, the existence of amoeba disease was reported in 1989 [18]. However, the study on its annual cycle that established its year-round existence in the local bees was done in 1998 [23]. Moreover, a nationwide series of diagnostic surveys conducted from 2008 to 2010 identified and located the amoeba disease of the honeybee in most places of the country [23, 24].

In Africa, the small hive beetle did not get attention in general and was considered as a minor pest for a long period. As a result, only little progress in the research has been made to determine its effect on the beekeeping industry in most places where it widely exists. This condition gave the pest sufficient time to widely spread in the continent and cause an undetermined reduction of beekeeping production. To this fact, the realistic effect of small hive beetles on Ethiopian honeybee colonies and bee products has not been yet investigated, despite its long-period distribution in different parts of the country. Moreover, no attempt has been made for the management of this pest to minimize its effect on the beekeeping industry in the country [25].

Both adults and larvae can be serious pests that weaken honeybee colonies or honey supers. The beetles multiply to vast numbers; and their larvae tunnel through the comb to eat brood, damage stored honey, and ultimately destroy infested colonies or cause them to abscond. The beetle also defecates in the honey, causing it to ferment and hence the honey runs out of the combs [8, 26]. In Ethiopia, the beetle was lately detected in 2000 in southern and southeastern parts of the country, including Teltele, Konso, Moyale, Segen, Moga, and Key Afer districts [27]. Subsequent studies show that the pests were found widely distributed in maize- and coffee-growing areas of the country [17, 24].

The identification and severity of each economically important honeybee pests and diseases have not been well documented in the study area. Detecting the occurrence and distributions of honeybees' health problems is a key step to prevent their harmful effects. Therefore, the study was carried out to generate baseline data on some common diseases of the honeybee colony in the Bonga district, which includes Gimbo and Chena sites of the Kaffa Zone of Southern Nations, Nationalities, and People's Region. This research was performed in the three districts of Kaffa Zone to assess the prevalence of major honeybee diseases.

## 2. Materials and Methods

### 2.1. Study Area

The study was carried out in the Bonga district of the Kaffa Zone of Southern Nations, Nationalities, and People's Region from October 2018 to March 2019 to determine the prevalence of honeybee diseases such as *Nosema(N. apis*) and amoeba (*M. mellificae*) diseases and pests such as small hive beetles. The altitudinal variation of the area ranges from 800 to 3300 m.a.s.l. and is divided into three major agroecological zones locally identified as Dega, Woyina-Dega, and Kola that are comparable to highland, midland, and lowland, respectively. Bonga is situated at a latitude and longitude of 7°16′N and 36°14′E, respectively. It is surrounded by the Gimbo woreda, the area that receives high rainfall with the average annual rainfall ranges from 1500 mm in the lowlands up to 2,000 mm at the highest elevations. Its annual average temperature ranges from 12.4°C to 26.8°C. Kaffa Zone with an area of 10,000 square kilometers has huge livestock resources; i.e. 921,964 cattle, 479,120 sheep, 241,256 goats, 79,438 horses, 10,870 mules, 148,626 colonies of the honeybee, and 2,226 donkeys [28, 29].

### 2.2. Study Design

A cross-sectional study was carried out from December 2018 to March 2019 in the three districts of Kaffa Zone on honeybee colonies managed under different beekeeping methods to investigate the prevalence of major parasitic diseases of honeybees. By observing and collecting samples from the colonies, the identification of pathogens causing bee diseases was done. Diagnoses were confirmed by integrating both clinical and parasitological studies.

### 2.3. Sampling Method and Sample Size Determination

A multistage stage sampling procedure was employed to select honeybee colonies. At the first stage, three administrative districts (Bonga, Chena, and Gimbo) were selected using purposive sampling based on their potential for beekeeping. In the second stage, three urban kebeles from Bonga and the two rural kebeles Kayikela and Qulish from Gimbo and Chena districts, respectively, were selected purposively based on their relative beekeeping potential and representing highland, midland, and lowland agroecologies. In the third stage, simple random sampling techniques were employed to select 128 bee colonies from each district with a total of 384 bee colonies. Since no previous such studies have been done in the area, the expected prevalence and absolute precision were set to be 50% and 5%, respectively. Based on this, the sample size was determined according to Thrusfield [30], which is as follows:where *n* is the required sample size, pexp is the expected prevalence, and *d* is the desired absolute precision.

(1) $$\begin{array}{c} n=\frac{1.96\;2\text{ pexp}\left(1-\text{pexp}\right)}{d2}, \end{array}$$

By using 50% expected prevalence with a 95% confidence interval at 5% absolute precision, the number of hives required to estimate prevalence was calculated to be 384.

### 2.4. Study Methodology

A single beehive was considered as one sample unit. Types of hive and agroecology were considered explanatory variables (risk factors) and tested whether they have an impact on the occurrence of honeybee diseases and pests. Honeybee hives were categorized as traditional, transitional, and modern hives. Three altitude categories were considered: highland (>2,400 meters), midland (1,800 to 2,400 meters), and lowland (<1,800 meters) above sea level [31].

#### 2.4.1. Data Collection Techniques

In order to examine the prevalence of infection of the abovementioned diseases and pests according to the activity periods of honeybees, samples were collected only one time—during the major honey flow season (November to February). Based on the observation for clinical symptoms, e.g., diarrhea in the beehive, adult bees were taken by brushing the bees off the comb through a large-mouthed funnel or directly in a universal bottle, and the bees were preserved in 70% ethyl alcohol and labeled accordingly. The samples were brought to the Bonga Agriculture Research Center Veterinary Laboratory. The occurrences of small hive beetles in the study areas were determined through hive inspection. The presence of a small hive beetle infestation (*A. tumida*) was identified through its adult, colony examination methods as described by Neumann et al. [32].

#### 2.4.2. Parasitological Investigation and Identification of the Parasitic Species

Examination of Nosema and amoeba diseases caused by a protozoan agent that affects the abdominal contents of adult honeybees was performed; sampling and diagnostic techniques were almost the same for both the diseases [33]. Therefore, following the procedure laid down by Fries et al. [34], 30–60 adult honeybees for a sample were collected from the hive entrance. The sample bees were collected in 70% alcohol until laboratory analysis. The abdomen of honeybees from each sample was cut using scissors. The cut abdomens were placed and grounded in a mortar containing 5–10 ml of tap water until an even suspension is formed using the pestle. The mortar and pestle were thoroughly cleaned before being used again. A loop of suspension was placed on a microscopic slide using the sterilized loop and covered with a cover slide. The suspension was examined under a microscope at 40× magnification power for the presence of Nosema spores and amoeba cysts.

### 2.5. Data Analysis

The data were entered and coded into Microsoft Excel and transferred to STATA software version 13 (Stata Corporation, College Station, USA) for statistical analysis. A chi-square test was used to assess the association of the risk factors with the prevalence of the major parasitic diseases of honeybees. Statistical significance was set at a *p* value of less than 0.05.

## 3. Results

### 3.1. Prevalence of *N. apis* and Associated Risk Factors

The current investigation revealed that the overall prevalence of *N. apis* in the Bonga district was 45.3% (Figures 1 and 2, Supplementary File). The prevalence of *N. apis* (67.96%) in highland, (39.84%) midland, and (28.12%) in the lowland was recorded. There was a statistically significant difference between these agroecologies (*p* < 0.006). The overall prevalence of *N. apis* has a significant variation between three types of hives (*p* < 0.02), and it was higher in traditional than both modern and transitional hive types. The highest prevalence of *N. apis* (63.5%) was observed in the traditional hive followed by transitional hive (43.54) (Table 1).

### 3.2. Prevalence of Amoeba (*M. mellificae*) and Associated Risk Factors

In the current study, the overall prevalence of amoeba (*M. mellificae*) (Figures 1 and 2, Supplementary File) disease was 40.3%. The result indicated that the honeybee amoeba (*M. mellificae*) disease was higher in traditional hives (51%) followed by transitional hives (41.62%) and modern hives (29.7%). Moreover, a statistically significant difference was observed between these hives (*p* < 0.032). The present results had also indicated that the amoeba (*M. mellificae*) disease was more common in highland areas (57%) than lowland (37.5%) and midland agroecologies (29.6%). Moreover, a statistically significant difference was observed between these agroecologies (*p* < 0.013) (Table 2).

### 3.3. Prevalence of Small Hive Beetles and Associated Risk Factors

The overall prevalence of small hive beetles (Figures 3 and 4, Supplementary File) is found to be 39.06%. In this study, the traditional hive was the most infested (54%) followed by the transitional hive (36.84%), while the modern hive is found to be the least affected (32.67%). There is a significant difference between types of hives (*p* < 0.028). For the prevalence of small hive beetles, there is also a significant difference regarding agroecology (*p* < 0.021). Lowland is found to be affected more (having 50.7%) followed by midland agroecology (37.5%) and highland (28.9%) (Table 3).

## 4. Discussion

In the current study, most of the sampled colonies for Nosema (*N. apis*) disease were found healthy and active in their duties. The same results were reported by Bezabeh [35] in the Central Highlands of Ethiopia [23]. This may be due to its low level of spore loads or increased out a flight for defecation due to the season or hygienic characters of bees. Yet, the impact of *N. apis* infection on beekeeping economics is enormous in a temperate climate, but it is often underestimated by beekeepers [36]. The disease is often referred to as the “Silent Killer” [37] because of the absence of obvious signs, and thus the disease is often not noticed. It retards colony development, thus affecting pollination and honey production, causing queen supersedure [38], and decreasing bee longevity [39]. An experiment conducted in Turkey by Yucel and Dogaroglu [40] proved that *N. apis* could give a very harmful effect on colony performance in the case of untreated honeybee colonies.

The overall prevalence of Nosema (*N. apis*) disease was 45.3%, which is in agreement with the study conducted by Yohannes et al. [41] who reported a 47% prevalence of Nosema disease in the Amhara region. However, it is little bit higher than that reported by Amssalu and Desalegn [23] who reported that 40.5% of the sampled honeybee colonies in the Southern Nations, Nationalities, and People's Region were infected by *N. apis.* Additionally, our finding is lower than those from other studies conducted in Addis Ababa by Begna and Kebede [6] who reported a prevalence of 53.3% and Yohannes et al. [41] who reported 58% in Oromia and 60% in Benishangul-Gumuz. This variation might be due to the sample collected season or the humidity difference of the geographical areas. It may also arise from differences in the hygienic behavior of the bee races.

The overall prevalence level of *N. apis* in highland (67.96%), midland (39.84%), and lowland (28.12%) was recorded. There was a statistically significant difference between these agroecologies (*p* < 0.006). This is not in agreement with Godifey [42] who reported highland, midland, and lowland overall prevalences of 50%, 31.6%, and 3.2%, respectively, in the Tigray region. This might be due to the effect of temperature and humidity that affect the spread of *N. apis*. The current finding is in agreement with the finding of Nega et al. [43] who stated that an increase in humidity and rainfall limit honeybees to fly out for cleansing, which in turn enhances the spread of the disease among the members and autoinfection.

The overall prevalence of *N. apis* has a significant variation between hives (*p* < 0.02), and it was higher in traditional than both modern and transitional hive types. The highest prevalence of *N. apis* was observed in the traditional hive (63.5%) followed by the transitional hive (43.54). Moreover, the modern hive is found to be the least affected (35.6%).

This result is not in agreement with the study result of Begna and Kebede [6] who stated *N. apis* disease was more prevalent in the modem beekeeping system (72.2%) than in the traditional (41.3%) and transitional (35.3%) systems This variation might be associated with the difference in the management practices like the placement of hive and changing of the frame. Additionally, traditional beehives are difficult to manipulate easily to control honeybee pests and diseases. As a result, they would become susceptible to pests and diseases.

Bezabeh [35] reported amoeba (*M. mellificae*) diseases were widely distributed and identified in most places of the country throughout the year. In our study, three hundred eighty-four (384) honeybee colonies were assessed for the presence of amoeba disease. The overall prevalence of amoeba disease was found to be 41.4% (*n* = 159). The result of the current study is lower than those of previous studies in different parts of the country such as the Oromia region with a prevalence rate of 88%, the Amhara region with 95%, and 60% in Benishangul-Gumuz [41].

This might be due to seasonal variation, as the amoeba infection is highly influenced by seasonal change since the current study was conducted around December and February where these were seasons of reduced rainfall. The result showed that *M. mellificae* pathogen occurred throughout the year. Moreover, a statistically significant difference was observed between these hives (*p* < 0.032). The current result disagrees with the finding of Begna and Kebede [6] who reported the prevalence of amoeba (*M. mellificae*) was high in the modern beekeeping system (88.9%) than in the traditional (61.9%) and transitional (47.1%) system. This may be due to differences in management practices, for instance, changing old combs, handling of equipment, and use of traditional control methods. The result also indicated that amoeba (*M. mellificae*) disease was more common on highland (57%) than lowland (37.5%) and the midland agroecology appeared to be affected the least (29.6%).

Moreover, a statistically significant difference was observed between these agroecologies (*p* < 0.013) which is not in agreement with the study reported by Begna and Kebede [6] who reported the prevalence of amoeba disease in highland, midland, and lowland was 85%, 52.3%, and 50%, respectively. This may be due to differences in weather conditions of different geographical locations. According to the result obtained by the current study, the prevalence of small hive beetles was found to be 39%. This is in agreement with the study conducted on 427 bee colonies located in 16 districts of south and southwest parts of the country, which revealed six districts and 43 bee colonies were positive to small hive beetles with the incidence ranging from 21% to 66% [17].

In this study, 54% of the traditional hive was infested with small hive beetles, followed by the transitional hive 36.84%, while the modern hive is found to be the least affected (32.67%). There is a significant difference between types of hives (*p* < 0.028). This may be attributed to the fact that traditional hives are more susceptible to the small hive beetle since these hives can easily harbor the pests; besides, traditional hives are more difficult to manipulate to control the pests and diseases unlike that of improved movable frame hives.

For the prevalence of small hive beetles, there is also a significant difference regarding agroecology (*p* < 0.021). Lowland is found to be affected more (having 50.7%) followed by midland agroecology (37.5%) and highland (28.9%). The causes of variation in prevalence among the studied districts may be attributed to different factors such as ecological variability, season, and management aspects.

Since these diseases have such a negative impact on hive products and colony development, and because they are common in the study area, fast control and prevention mechanisms are recommended, as well as the introduction of traditional control measures into modern beekeeping and vice versa. They are also needed for success in terms of product quality and quantity, as well as a healthy, disease-free colony. Due to time constraints, more research on the seasonal distribution and magnitude of honeybee diseases and pests, as well as their economic impact, is recommended, along with long-term colony monitoring for various types of organizations. Community or beekeeper's association extension programs, such as awareness building on issues related to bee diseases and its management and prevention methods, are required to be involved by either governmental or nongovernmental apiculture staff. In general, the beekeeping method has changed. Aside from that, the study area was one of the best places for beekeeping because of the variety of flowering plants.

## 5. Conclusions

The type of hive and agroecological zones are identified as risk factors for the presence of adult bee diseases, such as *N. apis* and amoeba (*M. mellificae*), and the pest small hive beetle in the study area. The study revealed that bees under the traditional type of hive are found to be more affected by the diseases and pests followed by transitional and modern hives. Regarding agroecologies, highland agroecology was the most affected by *N. apis*, while bees in lowland agroecology were found to suffer less from the disease. Different prevalence levels of *N. apis*, amoeba (*M. mellificae*), and small hive beetles were observed among the different study sites, agroecological zones, between apiaries, and types of hives. In order to classify and describe honeybee diseases and pests associated with Ethiopia's local honeybees, several field diagnostic surveys and laboratory research works need to be performed.

## Figures and Tables

**Table 1:** Prevalence of *N. apis* and associated risk factors.

Variables	Category	No. of sampled animals	Prevalence (%)	*X* ^2^	*p* value
Agroecology	Highland	128	87 (67.96%)	43.32	0.006
	Midland	128	518 (39.84%)		
	Lowland	128	36 (28.12%)		

Hive types	Modern	101	36 (35.6%)	13.97	0.02
	Transitional	209	91 (43.54%)		
	Traditional	74	47 (63.5%)		

**Table 2:** Prevalence of amoeba (*M. mellificae*) and associated risk factors.

Variables	Category	No. of sampled animals	Prevalence (%)	*X* ^2^	*p* value
Agroecology	Highland	128	73 (57%)	22.740	0.013
	Midland	128	38 (29.6%)		
	Lowland	128	48 (37.5%)		

Hive types	Modern	101	30 (29.7%)	8.619	0.03
	Transitional	209	87 (41.62%)		
	Traditional	74	38 (51%)		

**Table 3:** Prevalence of small hive beetles and associated risk factors.

Variables	Category	No. of sampled animals	Prevalence (%)	*X* ^2^	*p* value
Agroecology	Highland	128	37 (28.9%)	13.063	0.021
	Midland	128	48 (37.5%)		
	Lowland	128	65 (50.7%)		

Hive types	Modern	101	33 (32.67%)	9.152	0.028
	Transitional	209	77 (36.84%)		
	Traditional	74	40 (54%)		

## Data Availability

The data will be provided upon request to the corresponding author.

## Supplementary Materials

Supplementary Materials Figures showing the different parasitic species that were found during the research in the three districts of Kaffa Zone.
