# Supplementary figures and images for: Study on Major Parasitic Diseases of Adult Honeybees in Three Districts of Kaffa Zone, Southern Ethiopia
Source: Vet Med Int. 2021 Aug 11;2021:6346703. doi: 10.1155/2021/6346703 (PMC8376470; doi:10.1155/2021/6346703)

**
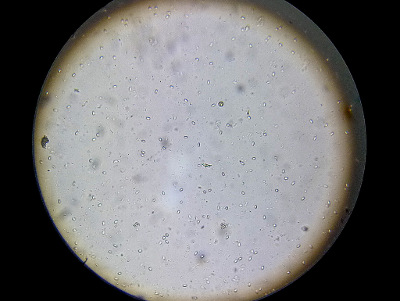

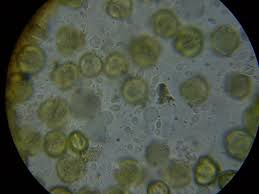
**

**Fig 1. Nosema spore Fig 2. Amoebae**

**
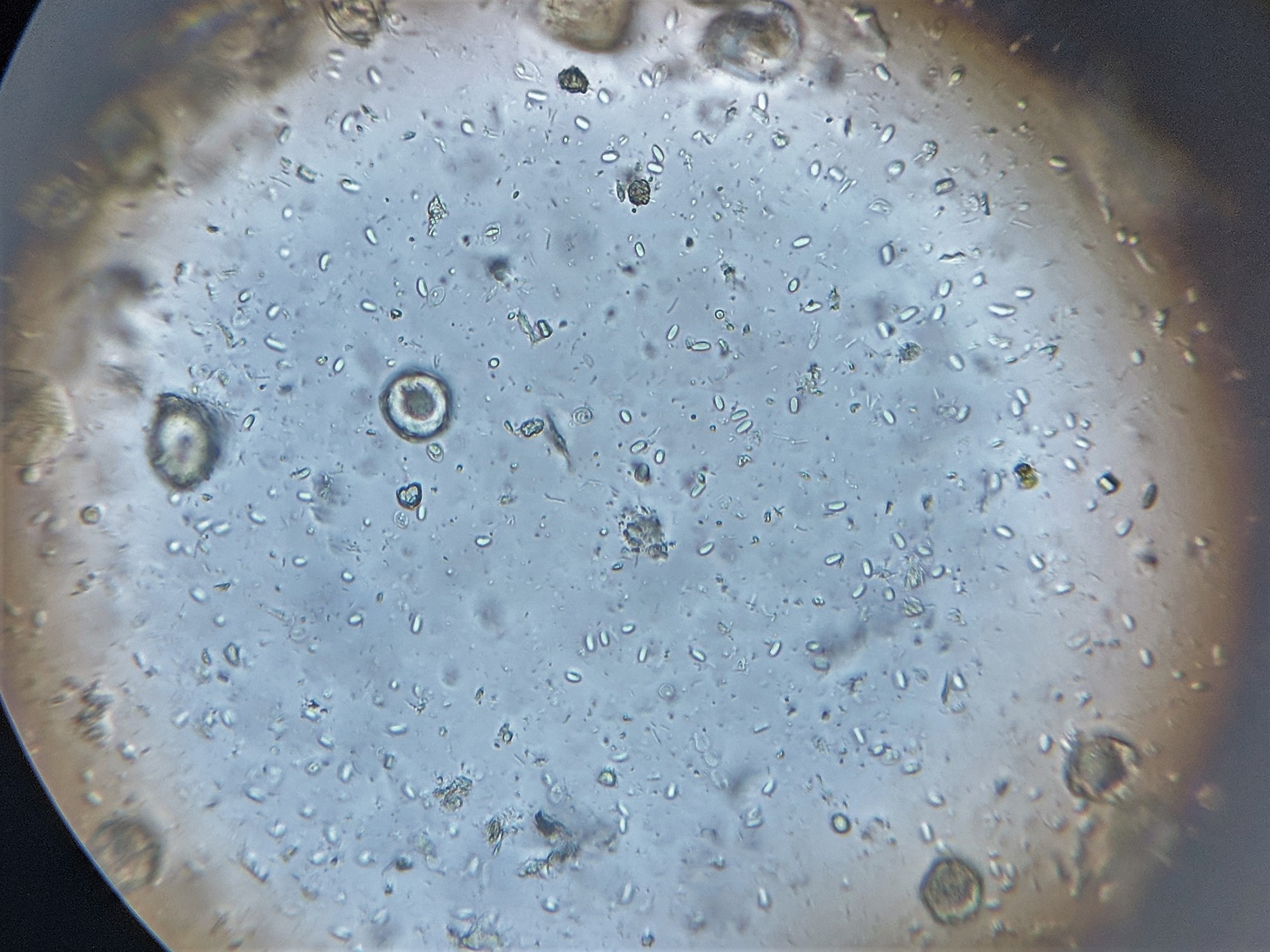

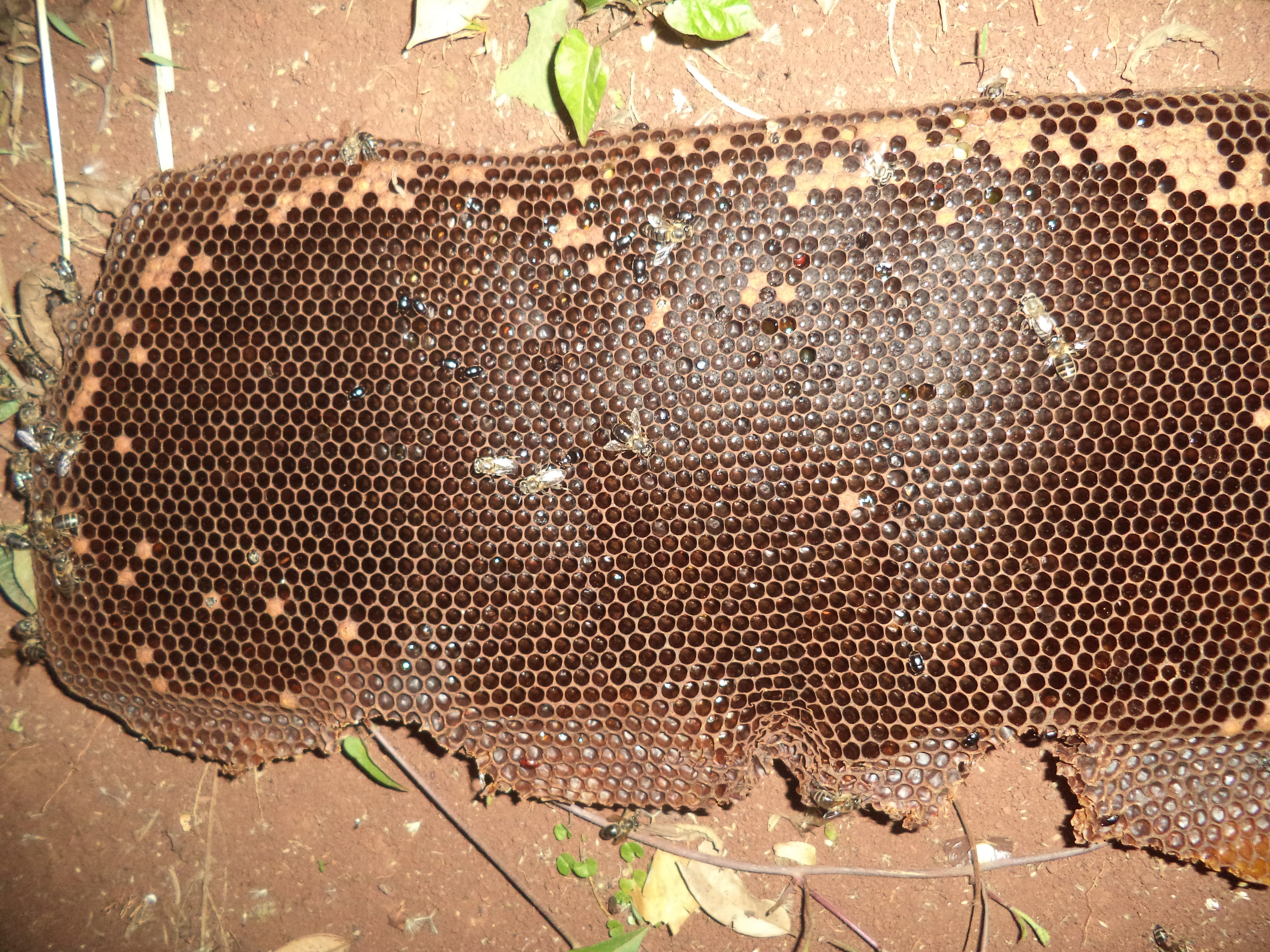
**

**Fig 3. Nosema and amoeba mixed infection. Fig 4. Small hive beetle**

Supplement: Supplementary Materials — Figures showing the different parasitic species that were found during the research in the three districts of Kaffa Zone. [file 6346703.f1.doc]
